# Visuomotor adaptation to constant and varying delays in a target acquisition task

**DOI:** 10.1167/jov.25.6.8

**Published:** 2025-05-23

**Authors:** Sam Beech, Danaë Stanton Fraser, Iain D. Gilchrist

**Affiliations:** 1 School of Psychological Science, University of Bristol, Bristol, UK; 2 Department of Psychology, University of Bath, Bath, UK

**Keywords:** visuomotor adaptation, delay adaptation, delayed visual feedback, visuomotor control

## Abstract

In visually guided movement tasks, visual feedback delays disrupt visuomotor control and impair performance. Adaptation then occurs as compensatory visuomotor updates are generated to accommodate the delay and recover control. Following the removal of the delay, an after-effect is observed, where the retention of this visuomotor update impairs post-exposure performance relative to the pre-exposure baseline. Although adaptation has previously been explored in response to constant delays, there has been no investigation into how continuously varying delays affect adaptation. In this experiment, participants completed a mouse-based target acquisition task with either a constant or varying delay between the mouse and cursor movements. At first exposure to the delays, completion times were large, and both delay conditions frequently overshot the target. With repeated exposure, the precision of the movements improved, resulting in lower completion times and fewer overshoots. The constant and varying delay conditions showed similar rates of change throughout the exposure phase, suggesting similar adaptation rates. Following the removal of the delay, the two delay conditions demonstrated similar post-exposure after-effects, as they systematically undershot the target and showed a decrease in overshooting relative to the pre-exposure baseline. Despite delay variability imposing an unstable error signal between the expected and actual cursor locations, this did not disrupt adaptation. These results suggest that the participants in the varying delay condition adapted to the mean delay and that the fluctuations away from this value did not disrupt the generation of the visuomotor updates.

## Introduction

Systematic perturbations to visual feedback cause a sharp decline in visuomotor control that is followed by steady improvement. This improvement is often attributed to visuomotor adaptation, an implicit learning mechanism that compensates for the perturbation through intersensory remapping (Mazzoni & Krakauer, 2006). Adaptation is often investigated in response to spatial displacements using prism glasses (Chapman et al., 2010) or virtual reality (Wright, 2014). In these paradigms, visual feedback is laterally shifted from the head orientation, and the participants are asked to point to a target. Initially, they point with an error magnitude equal to the spatial displacement, but over time, they adapt and learn to accommodate the displaced visual feedback to support reliable pointing. When the perturbation is removed, the participants show an after-effect as they now point in the opposite direction of the initial displacement. This after-effect demonstrates the learned compensatory update. Recently, research has begun to explore adaptation in response to temporal displacements, where a delay is placed between the motor action and the corresponding visual feedback. Delay adaptation with associated after-effects has been observed in a range of tasks (Botzer & Karniel, 2013; Cámara, de la Malla, López-Moliner, & Brenner, 2018; Cunningham, Billock, & Tsou, 2001a; Cunningham, Chatziastros, Von der Heyde, & Bülthoff, 2001; de la Malla, López-Moliner, & Brenner, 2012, de la Malla, López-Moliner, & Brenner, 2014; Foulkes & Miall, 2000; Kennedy, Buehner, & Rushton, 2009; Rohde, Van Dam, & Ernst, 2014) but only ever in response to constant delays that do not change throughout the movement. Investigating visuomotor control in response to delayed visual feedback is important as delays are common in a range of network-supported technologies, such as video games (Claypool & Claypool, 2006), virtual reality (Waltemate et al., 2016), and teleoperation systems (Sheridan, 1993). However, the delays in these applications are often unstable and continuously fluctuate (see Beznosyk, Quax, Coninx, & Lamotte, 2011). Therefore, it is important to determine how delay variability affects adaptation in a goal-directed visuomotor task.

Characteristic changes in the movement occur as participants adapt to constant visual feedback delays. At first exposure, participants cannot compensate for the delay when using vision to guide movement. Consequently, the movement timing is incorrect, as the participants start, update, and terminate their actions late. In tracking tasks, participants overshoot the apex of each turn as the change in direction is generated late (Rohde et al., 2014). In acquisition tasks, participants terminate their hand movement when they see their hand over the target, but as this feedback is delayed, they have already overshot the target by this point (Botzer & Karniel, 2013). With repeated exposure, the participants use the error between the expected and actual hand position to adapt, and their movements become increasingly precise (Rohde & Ernst, 2016). Delay adaptation is an anticipatory process within the vision-based feedback control mechanism as the participants learn to implicitly predict the non-delayed spatial state of the movement using an estimate of the delay, the lagged visual feedback, and the efferent feedback (Botzer & Karniel, 2013; Rohde et al., 2014). When the delay is removed, participants retain this predictive update, terminating the movement before observing their hand—or any controlled object—to have reached the target. However, as there is now no delay, the observed hand immediately stops, causing them to undershoot the tracking path apex (Rohde et al., 2014) or the target position (Botzer & Karniel, 2013). This post-exposure undershooting after-effect demonstrates how the delay is compensated during visually guided movement.

Although visuomotor adaptation in response to continuously varying delays has not been directly investigated, it has been proposed that the variability should disrupt adaptation due to the increased noise in the error signal (Rohde & Ernst, 2016). This hypothesis comes from temporal recalibration tasks, where perceptual updates are generated in response to a delay between a button press and a flash—these updates are generated faster when the delay between the button press and flash is held constant (Stetson, Cui, Montague, & Eagleman, 2006) in comparison to when it varies (Rohde & Ernst, 2013). The proposal that noise in the error signal disrupts adaptation is also consistent with the spatial adaptation literature. Wei and Körding (2010) tasked participants with guiding an unseen cursor to a target and provided them with spatially shifted visual feedback at the end of the movement. They varied the visual certainty between trials by adding large blur, small blur, or no blur to the visual feedback and found that reducing the certainty of the feedback reduced the adaptation rate. Therefore, delay variability may disrupt adaptation by increasing noise in the error signal, making it challenging to discern whether a given error was caused by an unpredictable delay fluctuation or an error in the visuomotor mapping that requires updating (see Wei & Körding, 2009).

However, one study investigating changes in performance across multiple sessions in a virtual reality shooting game reported similar improvements between constant and varying delay conditions. Beadle, Muth, and Pagano (2021) found that the two delay conditions showed similar improvements in their average time-to-hit scores over three sessions. However, adaptation could not be confirmed due to the absence of a post-exposure test for an after-effect. In general, poorer performance is observed under varying delay conditions (Beech, Stanton Fraser, Corston-Petrie, Gower, & Gilchrist, 2024; Davis, Smyth, & McDowell, 2010; Liu, Kwak, Devarakonda, Bekris, & Iftode, 2017), but it remains unclear whether this is due to a main effect of the delay condition or impaired adaptation.

In this experiment, we aimed to determine the impact of delay variability on adaptation by comparing performance between a constant (167 ms) delay condition and a varying (mean delay = 167 ms, jitter amplitude = 100 ms, frequency = 2.5 Hz) delay condition in a mouse-based target acquisition task. The task had three phases: a non-delay pre-exposure phase to set a baseline performance level, a delay exposure phase to measure the adaptation rate, and a non-delay post-exposure phase to test for an after-effect. Our preregistered hypothesis stated that delay variability would disrupt adaptation. We focused on completion time as the key performance measure, predicting that the varying delay condition would show a smaller decrease in completion time throughout the exposure phase. Typically, participants undershoot the target following adaptation (Botzer & Karniel, 2013), disrupting efficient target acquisition, which would lead to an increase in completion time from pre-exposure to post-exposure. Therefore, we hypothesized that the disrupted adaptation in the varying delay condition would result in a smaller increase in completion time from pre-exposure to post-exposure.

A formal hypothesis for overshooting was not included in our preregistration, but our general hypothesis was that delay variability would disrupt adaptation. Impaired adaptation would result in smaller effects in comparison to those reported following constant delay adaptation (e.g., Botzer & Karniel, 2013; Rohde et al., 2014). When first exposed to a constant delay, participants overshoot the target, but as they adapt, they produce increasingly accurate movements, and the number of overshoots declines. When the delay is removed, an undershooting after-effect is observed. Therefore, if delay variability disrupts adaptation, the varying delay condition will show a smaller decrease in overshooting throughout the exposure phase and a smaller decrease in overshooting from pre-exposure to post-exposure.

## Methods

The preregistration, task script, and data are available on the project OSF page: https://osf.io/ckrq2/?view_only=db5637fc948c4cc5a8238ad20d2fade2.

### Participants

Thirty-two adults (27 women, 5 men; aged 18–31) with corrected-to-normal vision completed the experiment. A further five did not complete the study (three due to poor eye-tracker calibration, one due to a mid-experiment fire alarm, and one due to self-reported illness) and were removed from analysis. The sample was split evenly, with 16 in the constant delay condition and 16 in the varying delay condition. All participants were students at the University of Bristol and received course credit for their participation. Ethical approval was obtained from the School of Psychological Science Research Ethics Committee, University of Bristol (12874).

### Apparatus and materials

Movements of the left eye were tracked at 1000 Hz using a video-based tower-mounted SR EyeLink 1000 (SR Research, Ottawa, Canada). The output was the horizontal and vertical gaze position coded in the display monitor's pixel coordinates. The head was kept stationary, and the chin rest was fixed 55 cm from the 24-inch ViewPixx (VPixx Technologies, Québec, Canada) monitor (60 Hz refresh rate, 1920 × 1080 spatial resolution, 0.277-mm × 0.277-mm pixel pitch). The experimental task was written in MATLAB R2021b (The MathWorks, Natick, MA, USA) using the Psychtoolbox-3 extension (Kleiner, Brainard, & Pelli, 2007). Participants used their dominant hand to operate a wired Razer (Razer, Irvine, CA, USA) Deathadder V2 gaming mouse that was hidden from view. The cursor movement was set to 1600 DPI, and the position was tracked at 60 Hz. The testing room was dark, with the ViewPixx display monitor as the sole light source.

### Baseline system latency

Due to data processing times, all computer systems have a baseline system latency between the user input and the corresponding on-screen update. A GoPro HERO13 was used to measure the baseline system latency in our setup using 400 frames-per-second slow-motion video. Across 10 recordings, the mean delay between the mouse and cursor movements was 35.8 frames, ranging from 34 to 38 frames. With a 400 Hz recording rate, this is a mean latency of 89.5 ms, with a range from 85 to 95 ms. This is a typical baseline latency (Hadjiosif, Abraham, Ranjan, & Smith, 2024) and reflects the baseline adapted state. All following latency values refer to additional latency beyond this baseline.

### Delay manipulation

The constant delay was experienced as a 167-ms lag between mouse and cursor movements. The native cursor was hidden from view, and its coordinates were stored for each frame. To achieve the 167-ms delay, the script extracted the cursor coordinates from 10 frames prior to the current frame (16.7-ms interframe interval at 60 Hz) and rendered a white circular cursor at this position. Delay variability was imposed by randomly selecting a new delay value within the 100-ms (six frames) amplitude range every 400-ms (2.5 Hz). As the delay was controlled in 16.7-ms increments (the interframe interval), a delay of 4 to 16 frames was randomly selected every 24 frames. During periods of smooth movement, the observed cursor would jump forward or backward along the movement path in response to decreases or increases in the delay, respectively. The observed cursor moved closer to the non-delayed position during periods of low latency and further from the non-delayed position during periods of larger latencies. Like jitter in network-supported technologies, there were instances when the cursor would disappear or “drop out” following large increases in the delay before updating to the new position. The 2.5 Hz frequency was chosen based on the values investigated in our previous work to minimize the occurrence dropout and flashing artifacts caused by large fluctuations (Beech et al., 2024).

### Task

**Figure 1.**
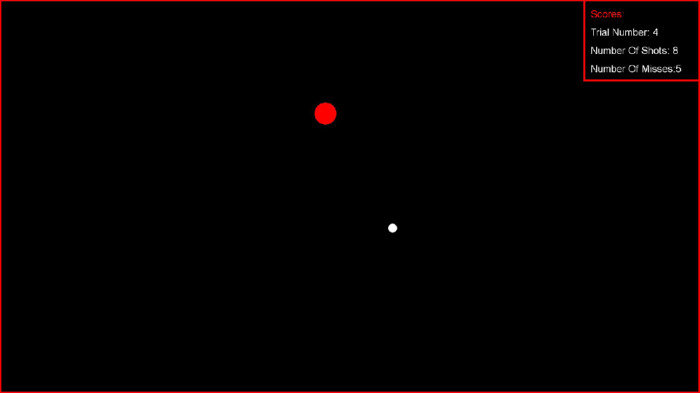
The task view. The red target remained static while the participants controlled the white cursor. The scores showed the trial number for the given block, the number of total shots (clicks), and a count of the misses.

The participants completed a target acquisition task with a series of static targets (Figure 1). At the trial onset, they were presented with a single red circular target (30 pixels/0.87 degrees of visual angle radius) in a randomized location within a 960-pixel × 960-pixel (27.2 × 27.2 degrees of visual angle) space. Their task was to place the center pixel of the white circular cursor (12.5 pixels/0.36 degrees of visual angle radius) within the target area and left-click the mouse. After a successful hit, the target disappeared and then reappeared in a new location after 267 ms. If the participants clicked while the center pixel of the cursor was outside the target area, the trial continued. Each block contained 15 targets (trials).

### Design

 The experiment had three phases. In the pre-exposure phase, the participants completed five non-delay blocks. In the exposure phase, they completed 32 blocks in either the constant or varying delay condition. In the post-exposure phase, the participants completed five non-delay blocks. The primary performance measure was the completion time, recorded as the time taken for the participant to complete the 15-trial block. Overshooting was recorded as the average number of trials per block where the cursor passed beyond the target, regardless of whether the participant clicked. This was a discrete measure, where the movement either overshot the target (1) or did not overshoot the target (0) within this trial. Therefore, the participants received a score out of 15 for each block, showing how many trials in that block had an overshoot. We also recorded the average cursor velocity as an exploratory measure to determine how the movement speed changed throughout the experiment.

Additional exploratory measures concerning the clicking accuracy and gaze behavior, which were not primary outcome variables, were also analyzed and are reported in the Appendix. Clicking accuracy (percentage of the total clicks per block that hit the target) was a key focus in our previous study (Beech et al., 2024). Therefore, these data were also recorded within the software for the present experiment. Previous research also shows that gaze behavior does not change when adapting to constant delays, with participants primarily fixating the moving target (Cámara et al., 2018). To investigate visual attention when adapting to constant and varying delays, we measured the average proportion of the trial spent fixating the target and the average number of fixations per trial for each block.

### Procedure

The eye tracker was calibrated using the standard 9-point calibration procedure. This was followed by a 9-point validation. A single round of calibration took 1 to 2 minutes. In cases where this validation was not classified as “good” by the EyeLink software (< 0.5 degrees of visual angle mean tracking error), the participants were recalibrated. If the validation was not classified as “good” on the second attempt, the participants were excluded from the study. If calibrated successfully, participants were informed that they would be playing a “shooting” game in which they had to click on a series of static targets. Participants were instructed to click on the targets as quickly and accurately as possible. This verbal instruction was given to prevent the participants from heavily prioritizing either accuracy or speed. During pilot testing for a previous study that used the same task (Beech et al., 2024), participants often excessively favored one metric—either moving very slowly and clicking only when settled on the target or moving very quickly and rapidly clicking as the cursor approached the target. Both behaviors prevent overshooting, removing a key error signal for delay adaptation. The inclusion of the verbal instructions effectively mitigated these behaviors and was therefore retained in this study.

The participants began each block by clicking in the center of the screen to initiate the first target. When they successfully clicked within the target area, the target disappeared and then reappeared in a new location after a 267-ms period—this was the upper bound of the jitter amplitude range and ensured that the cursor buffer was full before each trial. After successfully clicking on the final (15th) target, the block ended, and the participants were presented with a feedback screen showing their completion time (time to complete the block) and shot accuracy ((15 / total clicks) * 100). This was provided to maintain engagement and motivate improvement within the task. The participants could then begin the next block when they were ready by pressing the spacebar to close the feedback window and then clicking in the center of the screen to initiate the first target.

The participants were recalibrated at the beginning of the exposure phase and the post-exposure phase to maintain eye-tracking accuracy. The minimum calibration requirement also applied for these recalibrations. Upon progression to the post-exposure phase, the participants were informed that the delay had been removed. This is commonly done in delay adaptation research (e.g., Rohde et al., 2014) to prevent the retention of explicit strategies that could disrupt post-exposure performance and erroneously appear as an after-effect. It took 40 to 50 minutes to complete all three experimental phases. Finally, the participants completed a demographic questionnaire and were debriefed.

## Results

The exposure phase data were analyzed in JASP (JASP Team, 2023) using linear mixed-effects models (LMMs), with the “delay condition” as a categorical fixed effect, “block number” as a scalar fixed effect, and “participant” as a random-effects factor. The pre- to post-exposure changes were analyzed using a 2 (constant or varying delay) × 2 (pre-exposure or post-exposure) analysis of variance (ANOVA). The JASP software reported that none of our data sets violated the assumptions for the LMMs or ANOVAs.

### Completion time

**Figure 2.**
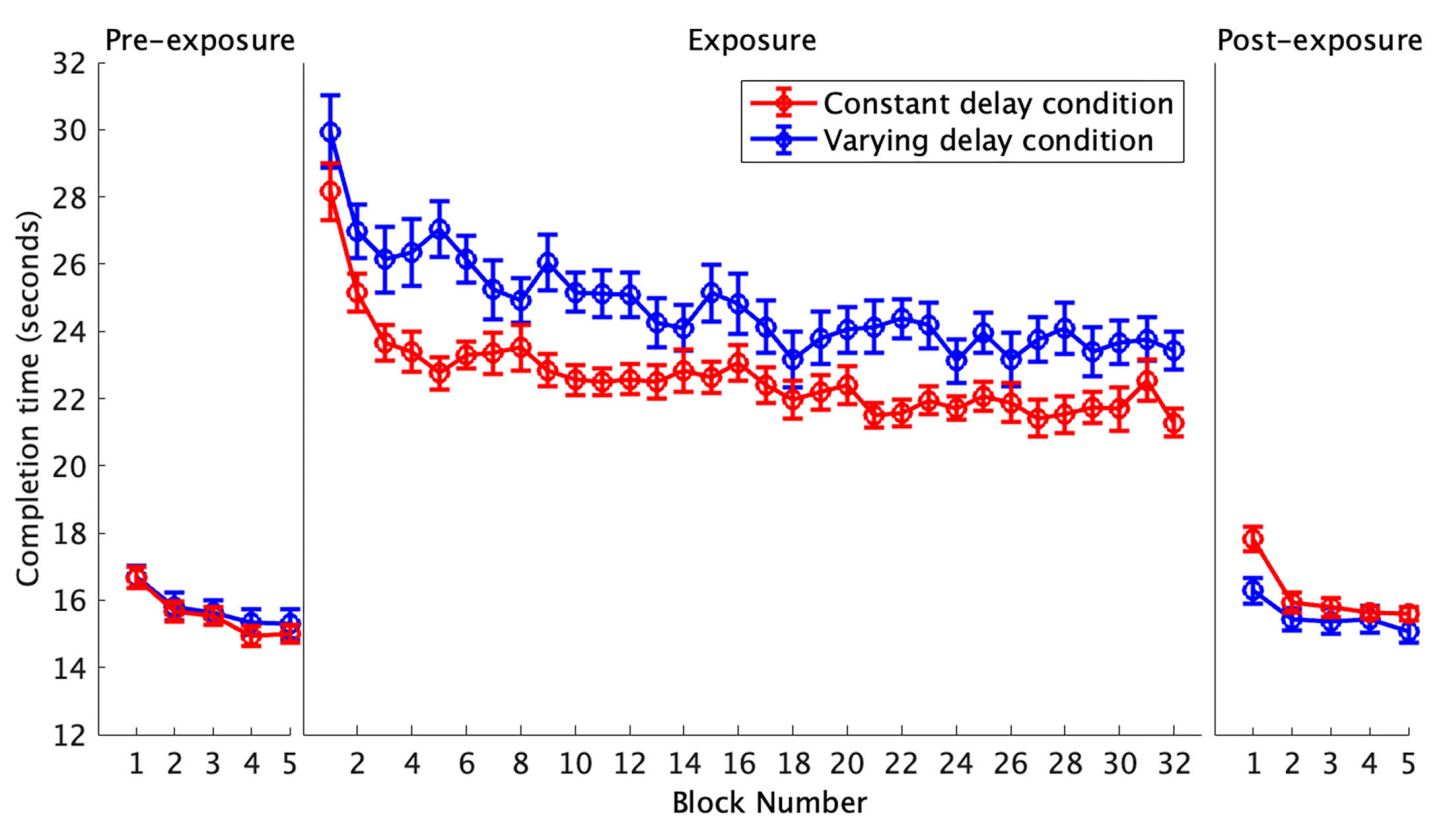
The average time taken to complete the 15-trial block. Each data point represents the mean completion time for each condition. The error bars show the standard error of the mean.

The mean completion times per block throughout the three experimental phases are presented in Figure 2. Both delay conditions demonstrated similar nonlinear trends throughout the exposure phase. Previous studies on visuomotor learning and adaptation have shown that the learning process is adequately modeled by an exponential function (e.g., Hosseini, Nguyen, & Joiner, 2017; Newell, Liu, & Mayer-Kress, 2001; Rashid, Kumari, Signal, Taylor, & Vandal, 2020). Although this was not preregistered, the block number was natural log transformed so that the trend became approximately linear for analysis in the LMM. The difference estimate between the delay conditions was significant, β = –1.34, *t*(30) = –2.47, *p* = 0.020, showing that the estimated marginal mean for the constant delay condition: 22.64 seconds, 95% CI [21.69, 23.60], was significantly lower than the estimated marginal mean for the varying delay condition: 24.77 seconds, 95% CI [23.81, 25.73]. The slope estimate for the block number was also significant, β = –1.47, *t*(30) = –11.09, *p* < 0.001, showing an overall decrease in completion time throughout the exposure phase. However, there was no evidence of an interaction, β = 0.11, *t*(30) = 0.83, *p* = 0.412. These results are consistent with previous research, where performance is poorer in varying delay conditions (e.g., Beech et al., 2024). However, both conditions demonstrated an improvement in completion time throughout the exposure phase, but there was no significant difference in the rate of change between the two delay conditions.

**Figure 3.**
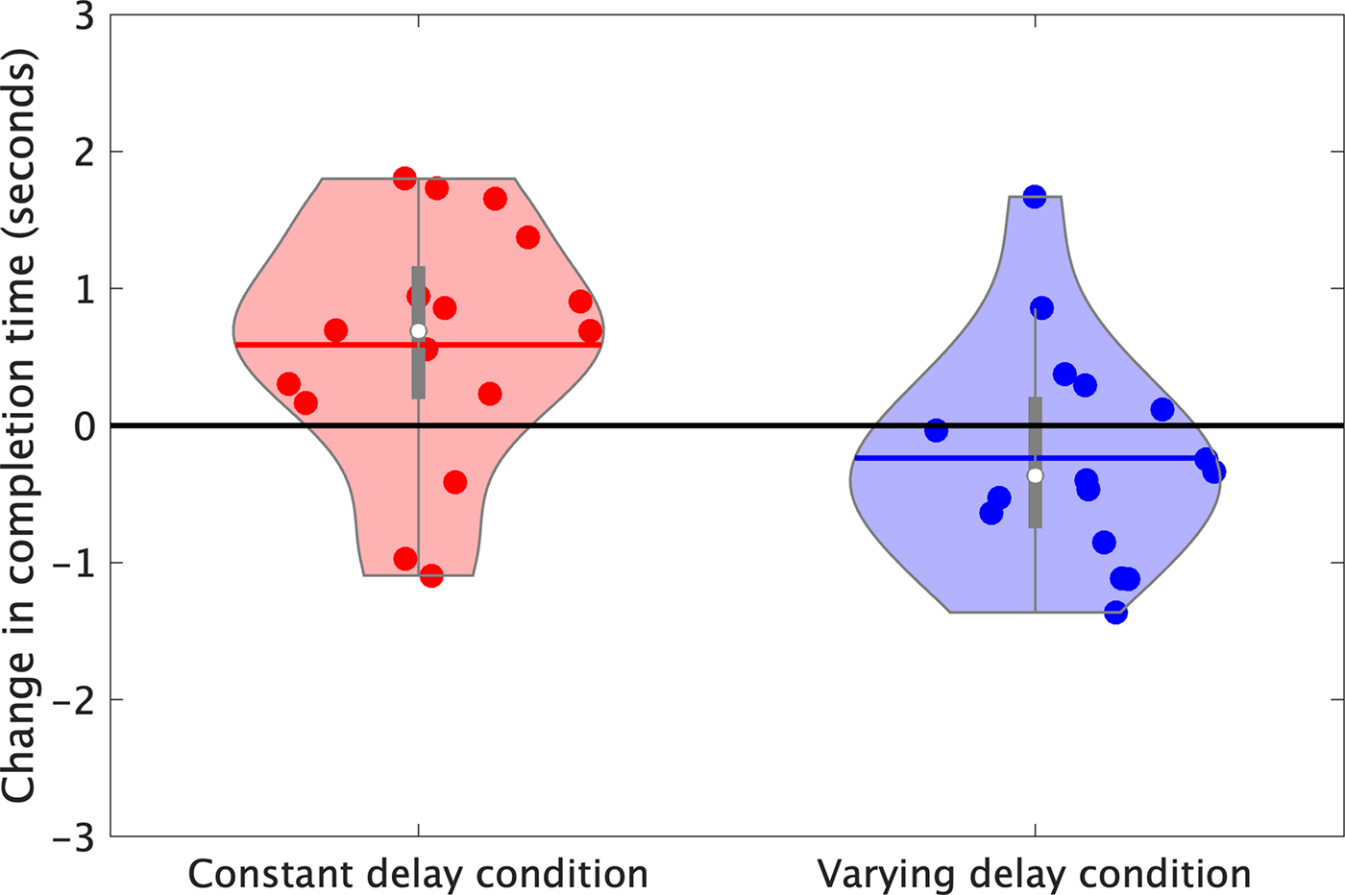
The change in the mean completion time per block from the pre-exposure phase to the post-exposure phase. The data points show each participant's change in mean completion time from pre-exposure to post-exposure. The mean and median are shown by the colored line and white dot, respectively. The thick portion of the gray line represents the interquartile range, and the whiskers represent the upper and lower quartiles. Values outside this range are outliers.

The change in the average completion time per block from pre-exposure to post-exposure was analyzed using a 2 × 2 ANOVA (Figure 3). Nonsignificant main effects were observed for the type of delay condition, *F*(1, 30) = 1.44, *p* = 0.240, *η*_p_² = 0.05, showing that the average completion times did not significantly differ between the constant (15.9 seconds) and varying (15.4 seconds) delay conditions. Furthermore, the main effect of the phase was nonsignificant, *F*(1, 30) = 0.36, *p* = 0.555, *η*_p_² = 0.01, showing no significant change in the average completion times from pre-exposure (15.7 seconds) to post-exposure (15.8 seconds). However, a significant interaction was observed, *F*(1, 30) = 7.90, *p* = 0.009, *η*_p_² = 0.21, showing that the post-exposure average completion time in the constant delay condition (16.1 seconds) was significantly larger than the varying delay condition (15.5 seconds).

### Overshooting

**Figure 4.**
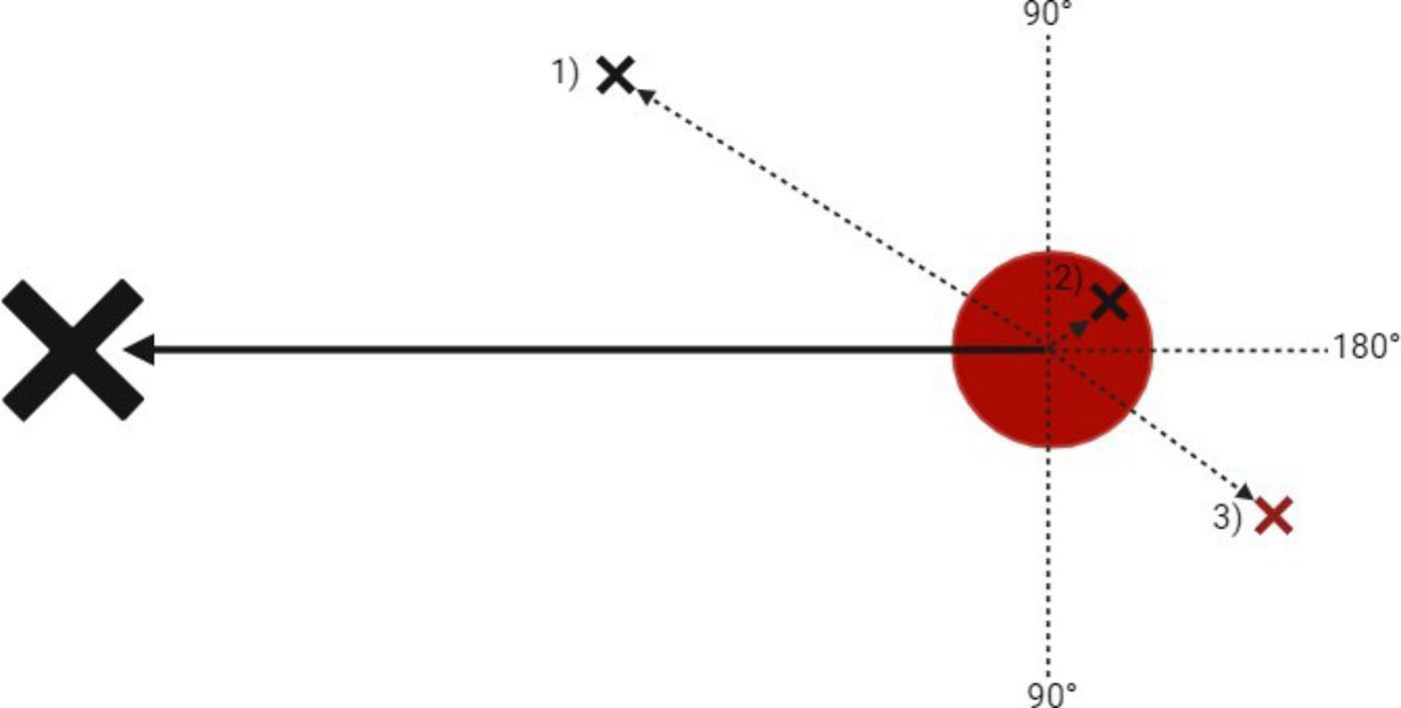
Overshoot calculation. A vector is calculated between the center of the target (red circle) to the cursor for the trial's first frame (large black cross). A new vector is then created from the target center to the cursor location for each frame, and the angular deviation between the two vectors is calculated. Cursor location 1 is an undershoot. Location 2 is not classed as an overshoot as it is past 90° but within the target boundary. Location 3 is an overshoot as it is past 90° and outside the target boundary.

Overshooting was recorded as the number of trials in a block with an overshoot. There were two criteria for a movement to be considered an overshoot (Figure 4). First, the absolute angular deviation of a current cursor-finish vector had to form a >90° angle with the start-finish vector. Second, the distance between the target center and the cursor position had to be greater than the target radius. This ensured that movements with an angular deviation greater than 90° that were within the target area were not classified as overshoots. A trial was classified as having an overshoot if any cursor position met these two criteria. For each block, the participants received a score from 0 (no trials with an overshoot) to 15 (all trials had an overshoot).

**Figure 5.**
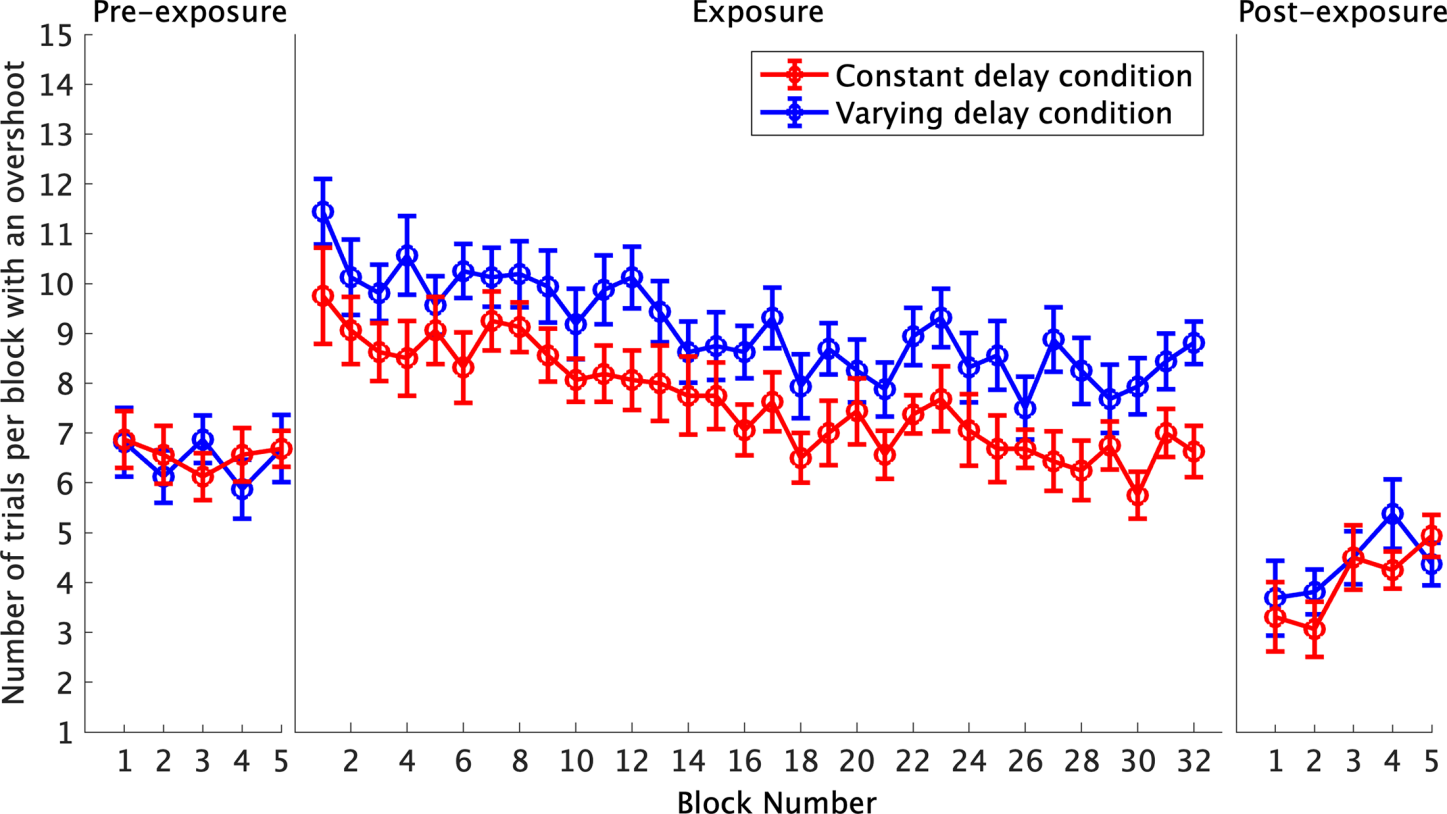
The average number of trials per block with an overshoot. Each data point represents the mean for each delay condition. The error bars show the standard error of the mean.

The change in the average number of trials per block with an overshoot throughout the exposure phase was modeled using an LMM (Figure 5). The difference estimate between the delay conditions was nonsignificant, β = –0.61, *t*(29.98) = –1.59, *p* = 0.123, suggesting no difference in the average number of overshoots between the two delay conditions. The slope estimate for the block number was significant, β = –0.09, *t*(29.99) = –8.91, *p* <.001, showing a decrease in overshooting as the block number increased. There was no evidence of a two-way interaction, β = –0.01, *t*(29.99) = –0.73, *p* = 0.472, demonstrating no difference between the two delay conditions in the rate of change in overshooting throughout the exposure phase. Despite the absence of a significant main effect of the type of delay condition, visual inspection of Figure 5 shows a difference in the average number of overshoots between the two delay conditions. This suggests that the planned statistical test was not appropriate for detecting this difference. Both conditions demonstrated a significant decrease in overshooting throughout the exposure phase that was consistent with adaptation, but the rates of change did not significantly differ between the two delay conditions.

The change in the average number of trials per block with an overshoot from pre-exposure to post-exposure was investigated using a 2 × 2 ANOVA. The main effect of phase was significant, *F*(1, 30) = 71.94, *p* < 0.001, *η*_p_² = 0.71, showing a significant decrease (–2.34) from pre-exposure (6.52) to post-exposure (4.18). The main effect of the delay condition, *F*(1, 30) = 0.06, *p* = 0.814, *η*_p_² = 0.002, and the interaction, *F*(1, 30) = 0.60, *p* = 0.447, *η*_p_² = 0.02, were both nonsignificant. This analysis shows a similar decrease in overshooting from pre-exposure to post-exposure for both delay conditions.

### Cursor velocity

The position of the observed cursor was recorded and stored for each frame. As previously noted, there were periods in the varying delay condition where the cursor position could briefly drop out following large fluctuations, leading to null values in the data for the cursor position. For these periods, a Piecewise Cubic Hermite Interpolating Polynomial (PCHIP) method was used to produce estimates for the cursor position that preserve the shape and direction of movement. We then used the central difference method to calculate the interframe velocity for each consecutive position in pixels per frame. These values were then converted to meters per second using the known pixel pitch of the ViewPixx monitor (0.277 × 0.277 mm) and the 60 Hz interframe interval (16.7 ms).

**Figure 6.**
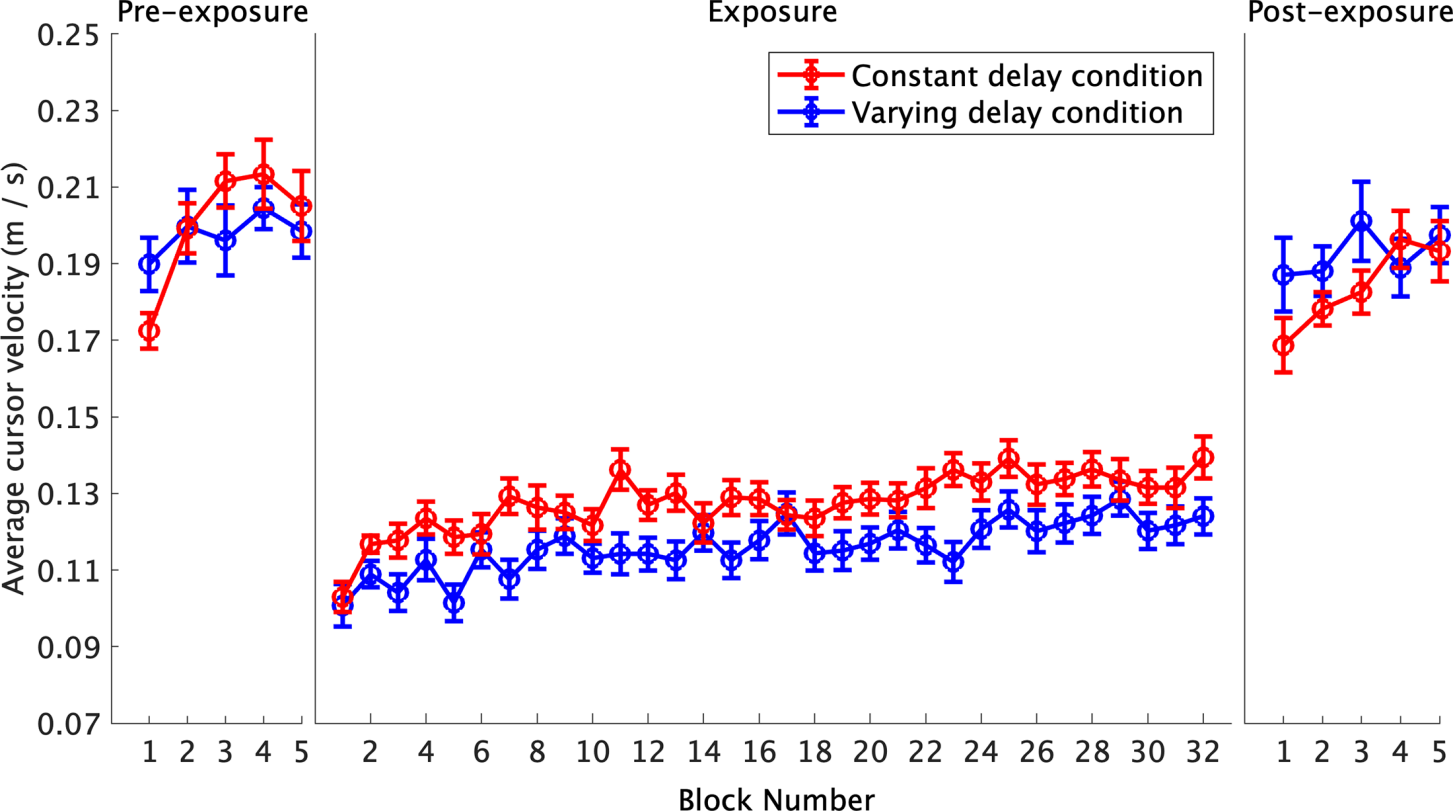
The average cursor velocity. Each data point represents the mean cursor velocity for each delay condition. The error bars show the standard error of the mean.

The change in the average cursor velocity per block throughout the exposure was investigated using an LMM (Figure 6). The difference estimate between the delay conditions was significant, β = 0.005, *t*(28.41) = 2.30, *p* = 0.029, showing that the estimated marginal mean for the constant delay condition, 0.128 meters per second, 95% CI [0.121, 0.134], was larger than the estimated marginal mean for the varying delay condition, 0.116 meters per second, 95% CI [0.109, 0.123]. The cursor velocity in the constant delay condition was, on average, faster by just over 1 cm per second. The slope estimate for the block number was also significant, β = 0.0006, *t*(29.91) = 8.48, *p* < 0.001, showing an increase in the average cursor velocity throughout the exposure phase. However, there was no evidence of an interaction, β = – 0.00003, *t*(29.91) = –0.39, *p* = 0.697, demonstrating no difference between the two delay conditions in the rate of change in velocity throughout the exposure phase.

The change in the average velocity from pre-exposure to post-exposure was investigated using a 2 × 2 ANOVA. The main effect of phase was significant, *F*(1, 30) = 6.40, *p* = 0.017, *η*_p_² = 0.18, showing a significant decrease (−0.01 m per second) from pre-exposure (0.20 m per second) to post-exposure (0.19 m per second). However, the main effect of the delay condition, *F*(1, 30) = 0.26, *p* = 0.614, *η*_p_² = 0.01, and the interaction, *F*(1, 30) = 2.05, *p* = 0.163, *η*_p_² = 0.06, were nonsignificant. Figure 6 shows differences in the early post-exposure movement speed that are consistent with our hypothesis, with faster movements in the varying delay condition. However, the expected interaction was nonsignificant.

### Movement profiles

**Figure 7.**
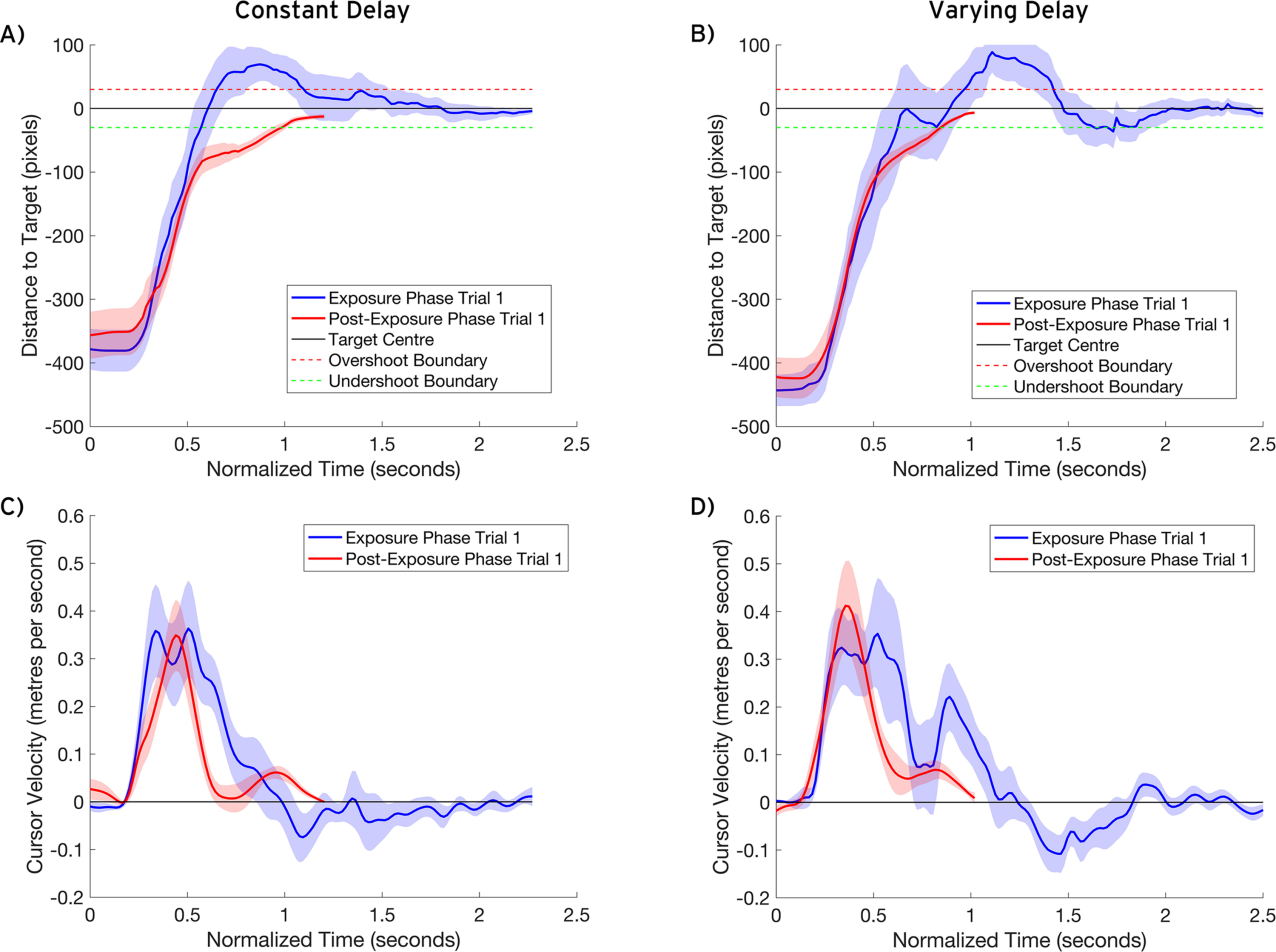
The average cursor-target distance profiles and velocity profiles for the constant delay condition (A, C) and the varying delay condition (B, D). The profiles were normalized to the mean trial length for the given phase for each delay condition. Trials shorter than the mean were interpolated using PCHIP to preserve the movement trajectory and retain the features of the original path. Trials longer than the average were downsampled using PCHIP by remapping the original time series onto the normalized trial duration, ensuring key trajectory features were preserved. The shaded areas represent the standard error.

Profiles showing the average cursor-target distances and velocities throughout the movements are presented in Figure 7. Botzer and Karniel (2013) reported the average movement profiles for their catch trials, where participants were either suddenly exposed to a constant delay following a period of no delay, resulting in overshooting (delay catch), or experienced a no-delay trial after adapting to a constant delay, resulting in undershooting (no-delay catch). For direct comparison, we present the average profiles for the first exposure phase trial to reflect the delay catch trial and the average profiles for the first post-exposure trial to reflect the no-delay catch trial. The movement profiles are consistent with those reported by Botzer and Karniel (2013). At first exposure to the delay, the participants overshot the target and showed negative velocity as they moved their cursor back toward the target. However, following adaptation, the removal of the delay led to undershooting, with a second peak in velocity.

## Discussion

This experiment investigated the impact of delay variability on adaptation in a mouse-based target acquisition task. The adaptation rate was measured using the rate of change in performance throughout the exposure phase. After-effects were measured using the change in performance from pre-exposure to post-exposure. We predicted that delay variability would impair adaptation, resulting in smaller changes in performance throughout the exposure phase (slower adaptation rates) and smaller changes from pre-exposure to post-exposure (smaller after-effects) compared to the constant delay condition. However, our results did not support this hypothesis. Throughout the exposure phase, completion time decreased, overshooting decreased, and the cursor velocity increased, but there were no significant differences in the rates of change between the two delay conditions. When exploring the pre- to post-exposure change in completion time, we observed a significant interaction, with Figure 2 showing that the post-exposure completion time was shorter following exposure to the varying delay. While consistent with our hypothesis, this difference was small and not observed in other measures—both conditions showed significant decreases in cursor velocity and overshooting from pre-exposure to post-exposure, but the interactions were nonsignificant. Additionally, the average movement profiles for the first post-exposure trial portray similar undershooting after-effects in both delay conditions (Figure 7). The similar adaptation rates and after-effects between the two delay conditions show that delay variability did not disrupt adaptation within this task.

To our knowledge, this experiment provides the first example of adaptation to a continuously varying delay with an associated after-effect. This finding builds upon the work of Beadle et al. (2021), who reported similar improvements between their constant and varying delay conditions across three sessions in a virtual reality target acquisition task. They proposed that the participants adapted to the constant and varying delays, but this could not be verified due to the absence of a post-exposure test for an after-effect. Within our task, evidence of adaptation was observed in both conditions with after-effects in all three measures. Furthermore, as Beadle et al. (2021) did not explore the rate of change in performance within each session, learning rates could not be compared between the two delay conditions. Throughout the exposure phase, we observed similar changes in completion time, cursor velocity, and overshooting for both delay conditions, indicating similar adaptation rates. Therefore, our results support the conclusion presented by Beadle et al. (2021) and show that delay variability did not disrupt the rate of adaptation.

However, the absence of adaptation impairments in the varying delay condition was unexpected as increasing the noise in the error signal has been observed to impair spatial adaptation (Wei & Körding, 2010) and perceptual recalibration (see Rohde & Ernst, 2016). We propose two explanations as to why the delay variability did not disrupt adaptation in our task. First, in comparison to the spatial adaptation and perceptual recalibration paradigms, the frequency of exposure to the error signal was much greater in our task. In the spatial adaptation task, perception of the error signal was limited to 100 ms at the end of each movement (Wei & Körding, 2010). In temporal recalibration paradigms, participants momentarily experience the delay between the button press and flash. In contrast, our participants continuously perceived the delay between the mouse and cursor as they executed their movements. By the end of the first block, they had experienced ∼30 seconds of continuous delay fluctuation at 2.5 Hz, exposing them to ∼75 changes in the delay. This increased frequency of exposure to the fluctuating error signal may have allowed the participants to filter the noise and identify the mean delay at a much faster rate. Second, Knelange and López-Moliner (2019) show that adaptation is disrupted when the noise in the error signal is nonstationary, where the statistical properties such as the mean and variance change over time. However, the varying delay within our task had stationary characteristics, with a clearly defined mean, equidistant positive and negative amplitude poles, and a uniform distribution. Models of adaptation show that learning is optimal when the noise has stationary statistics, such as Gaussian noise (Burge, Ernst, & Banks, 2008). Therefore, the stationary characteristics of the noise within the varying delay condition in the present task may have meant that the fluctuations away from the mean delay did not disrupt the generation of the visuomotor updates.

Delay adaptation is characterized by post-exposure undershooting, meaning that the observed decrease in overshooting from pre- to post-exposure must be considered in relation to the cursor-target distance (Figures 7A, B) and velocity profiles (Figure 7C, D). In isolation, the decrease in overshooting does not conclusively demonstrate a post-exposure undershooting after-effect. Instead, this could reflect increased accuracy in the movement. However, if this were the case, we would not expect the participants to show an increase in overshooting throughout the post-exposure phase back toward the pre-exposure baseline. Importantly, the movement profiles are consistent with those reported by Botzer and Karniel (2013). In the first exposure phase trial, the cursor moves past the target, and the negative velocity shows the movement back toward the target. In the first post-exposure trial, the cursor undershoots the target, and there is a second peak in velocity as they generate the second movement toward the target. These profiles are similar between conditions, showing that the constant and varying delay condition generated similar visuomotor updates.

Visual inspection of the movement profiles reveals how the online control system responds at first exposure to the delays (Figure 7). Specifically, we observe instances of online corrections at two key points in the movement. First, there are dual velocity peaks at ∼300 ms and ∼500 ms. The ∼175-ms interval between the movement onset and the first peak exceeds the 90- to 120-ms latency of online corrections observed in response to sudden visual disruptions (Cross, Cluff, Takei, & Scott, 2019), suggesting that these dual peaks do not reflect a reactive correction to the initial perception of the delay. Alternatively, they may reflect an online correction in response to the spatial discrepancy between the expected and actual cursor positions at the first velocity peak. In standard reaching movements, a bell-shaped velocity profile is observed, with movement features such as the peak velocity being preplanned and influenced by the start-finish distance (Messier & Kalaska, 1999). In the present task, the delayed cursor lags the expected, non-delayed position at the first peak. Consequently, the participants may have responded with a corrective increase in velocity to bring the delayed cursor closer to the expected position, resulting in the second peak before the deceleration period. A second notable feature of the movement profiles is that the deceleration period is not smooth as the cursor approaches the target at ∼750 ms. This may reflect interference from the forward model prediction, where the delayed cursor is expected to be at the target as this point in the movement. Consequently, the participants decrease the movement speed, but as the delayed cursor has not yet reached the target, they then increase the movement speed again. These effects are larger for the varying delay condition, and these points in time coincide with the changes in the delay (every 400 ms). The differences in the profiles for each delay condition suggest that the delay fluctuations have a large impact on the control of the movement. The change in participants’ responses to delay fluctuations as they adapt presents an intriguing direction for future research, offering insight into how they learn to manage the fluctuations. However, we acknowledge that the limited sample for these profiles may have inflated these differences. While the PCHIP method preserves the shape of the true movement, movements of different distances will naturally have different peak velocities and profiles (Cieślik & Łopatka, 2022; Messier & Kalaska, 1999; Nagasaki, 1989). To investigate changes in movement kinematics as participants adapt to varying delays, future research should adopt the approach used by Botzer and Karniel (2013), in which the start-target vector and movement velocity are standardized across trials.

Despite similar adaptation rates and after-effects, significant main effects of the delay condition were observed. The varying delay condition demonstrated slower average completion times and slower average cursor velocities. This is consistent with previous research, where poorer performance is typically observed under varying delay conditions (Beech et al., 2024; Davis et al., 2010; Liu et al., 2017). However, it has remained unclear whether this is due to impaired adaptation or the increased difficulty in controlling movements with an unpredictably fluctuating visual feedback delay. Our results support the latter explanation. Even after adapting, it remains challenging to control a delayed cursor, an effect that was even more pronounced in the varying delay conditions. For both conditions, average completion times were noticeably longer and movement velocities lower during the exposure phase in comparison to the two non-delay phases (Figures 2 and 6). Such an effect is often observed in response to delayed visual feedback in teleoperation applications as the user slows down to recover visuomotor control (Ferrell, 1965; Sheridan, 1993). Consequently, the poorer average performance within the varying delay condition could be attributed to the increased difficulty in controlling a cursor that is constantly jittering during smooth movements. Slowing down a movement in response to delayed visual feedback has been proposed to impair adaptation (Rohde & Ernst, 2016), but Cunningham, Chatziastros, et al. (2001) found that the middle of three speeds resulted in the largest adaptation in their driving simulator task. Botzer and Karniel (2013) demonstrated that participants can adapt when they are in control of the movement speed. However, a direct comparison of adaptation rates between participant-controlled and task-controlled movement speed conditions has not yet been conducted, and it remains unclear which approach is optimal.

An additional notable finding was the significant interaction in the completion time data, indicating that participants in the constant delay condition exhibited larger post-exposure completion times. While this effect is consistent with our hypothesis, the absence of significant differences in the adaptation rates between the two delay conditions suggests that this was not driven by differences in adaptation. In the post-exposure phase, both delay conditions demonstrated similar undershooting, suggesting similar movement paths. Therefore, the absence of a significant pre- to post-exposure increase in completion time for the varying delay condition may indicate that they were better prepared to react to the sensory prediction errors following the removal of the delay. In the constant delay condition, precise estimates of the non-delayed spatial state of the movement can be learned as the delay remains fixed. In contrast, the participants in the varying delay condition can only estimate the mean as best guess for the delay and must be prepared to react to unexpected jumps in the cursor position. This may have prepared them to efficiently respond to the post-exposure errors, reducing their impact on completion time. The average movement profiles support this conclusion (Figure 7), as the varying delay condition does not reach zero velocity before the second peak when correcting for the undershoot and shows a smoother approach to the target. Figure 6 also provides support for this hypothesis, with the varying delay condition showing slightly greater average velocities in the first post-exposure trial. However, there were no significant differences in the post-exposure velocity between the two delay conditions, so this conclusion is not statistically supported.

The exploratory eye-tracking analysis (see Appendix) was consistent with previous research concerning visual attention during delay adaptation. Cámara et al. (2018) found that eye movements do not change when adapting to delayed visual feedback, with participants largely fixating a moving target and guiding the cursor toward it using peripheral visual feedback (Cámara et al., 2018). The eye-tracking data show that the participants spend most of the trial fixating the target (∼80%). The remaining 20% encompass the start of the trial, where they must visually locate the target and other instances where they may need to visually relocate the cursor due to loss of control. Although the target fixation proportion decreased throughout the exposure phase, this was likely due to the decreasing time taken to complete the trial. The time taken to fixate the target from trial onset would remain consistent, but the period spent fixating the target would decrease as they acquired the target with greater speed, leading to a lower target fixation proportion. This is supported by the absence of a significant change in the average number of fixations per trial throughout the exposure phase, suggesting little change in visual behavior. We also observed no after-effects, further demonstrating that gaze behavior does not change when adapting to delays. Our results support the findings from Cámara et al. (2018), showing that visual behavior did not change when adapting to the delays.

We initially stated that delay adaptation occurs through an implicitly learned estimate of the delay signal (see Rohde et al., 2014), but the way in which the brain represents and compensates for visual feedback delays remains uncertain. The time-based explanation proposes that delay adaptation is achieved through the integration of an implicitly learned delay estimate, the efferent motor command, and the observed visual feedback to predict the current spatial state of movement (Rohde et al., 2014). This is supported by undershooting of the target following the removal of the delay. In contrast, the state-based explanation posits that the error between the expected and actual position is represented as a change in spatial gain (Avraham et al., 2017). This is evidenced by post-exposure overshooting when movements are completed without visual feedback. While our findings align with the time-based explanation, it is possible that we may have instead found support for the state-based explanation had the post-exposure phase been conducted without visual feedback. However, Avraham et al. (2017) offer an explanation that integrates these two hypotheses. They propose that shorter delays, such as those under 100 ms within their tasks, are represented spatially, whereas longer delays are represented temporally. In our task, the delays—excluding the 89.5-ms mean baseline system latency—were 167 ms for the constant delay condition and 67 to 267 ms for the varying delay condition. Therefore, it is likely that they were represented temporally. State-based representation of delays becomes particularly intriguing when considering instances in which visuomotor delay adaptation is accompanied by perceptual recalibration. For example, Cunningham, Billock, et al. (2001) noted that after adapting to a 235-ms delay in an obstacle avoidance task, their participants spontaneously reported that the delayed visual feedback became perceptually synchronous with their actions. When the delay was removed, they also reported an illusory reversal of cause and effect, where the on-screen outcome was perceived to occur before their action. Given that perceptual recalibration requires a time-based representation for such an effect to occur, a potential dichotomy between state-based visuomotor updates and time-based perceptual recalibration presents an interesting direction for delay adaptation research.

We wish to highlight a limitation of our experiment that prevented us from comparing the peak velocities between the delay conditions. Specifically, cursor velocities were calculated based on the position of the delayed cursor, rather than the hidden, non-delayed cursor. We initially believed this was a better approximation of the movement as it reflected what the participant observed. However, this introduced complications when recording the cursor positions for the varying delay condition. Periods of cursor dropout due to large delay increases were manageable, as missing data could be accurately interpolated using PCHIP. However, large changes in the delay occasionally caused the delayed cursor to jump position within a single frame. Specifically, large decreases in the delay would cause the cursor to jump closer to the non-delayed position in a direction consistent with the ongoing movement. Consequently, this artificially increased the peak velocity for that movement. Importantly, this issue had a minimal impact on average velocity across the trial and did not lead to a false negative in our analysis, as the constant delay condition still demonstrated a significantly greater average velocity. It also did not affect the overshoot analysis, since the delayed cursor could only move to a position previously occupied by the true cursor. Nonetheless, this prevented meaningful analysis of the peak velocity, as movements in the varying delay condition could appear to have artificially high peaks within a single frame. In future experiments using this task, we will ensure that the cursor position is recorded from the hidden, non-delayed cursor.

## Conclusions

The results show that delay variability did not disrupt adaptation in our mouse-based target acquisition task. The participants in the constant and varying delay conditions demonstrated similar adaptation rates throughout the exposure phase. They also exhibited similar after-effects, through pre- to post-exposure decreases in overshooting and average cursor-target distance profiles and velocity profiles that showed a tendency to undershoot the target. This finding has important implications for understanding how noise in the error signal can be filtered, as the participants were able to efficiently adapt to the mean delay. Our results show that delay adaptation is unaffected by a varying delay condition with stationary noise characteristics.

## Appendix

### Accuracy

Overall accuracy was very high, with 92% for the constant delay condition and 90% for the varying delay condition. This suggested that ceiling effects were present for both conditions, so no formal analysis was performed.

### Target fixation proportion

The change in the average proportion of the trial spent fixating the target throughout the exposure phase was analyzed using an LMM. All blinks were removed from analysis before this calculation. The difference estimate between the delay conditions was nonsignificant, β = –0.0003, *t*(30) = –0.04, *p* = 0.967, showing no significant difference in the average target fixation proportion between the constant delay condition, 79.6%, 95% CI [77.6%, 81.5%], and the varying delay condition, 80.5%, 95% CI [78.5%, 82.4%]. The slope estimate for the block number was significant, β = –0.0006, *t*(30) = –3.42, *p* = 0.002, showing a decrease in the target fixation proportion as the block number increased. However, there was no evidence of an interaction, β = 0.0002, *t* = –1.40, *p* = 0.171. The change in the average proportion of the trial spent fixating the target from pre-exposure to post-exposure was investigated using a 2 × 2 ANOVA. The main effect of the phase was nonsignificant, *F*(1, 30) = 0.28, *p* = 0.598, *η*_p_² = 0.01, showing no significant change from pre-exposure (73.4%) to post-exposure (73.9%). The main effect of the delay condition was nonsignificant, *F*(1, 30) = 0.25, *p* = 0.622, *η*_p_² = 0.01, showing no significant differences between the constant (73.4%) and the varying delay condition (73.9%). The interaction was also nonsignificant, *F*(1, 30) = 0.004, *p* = 0.951, *η*_p_² = 0.0001.

### Number of fixations

The change in the average number of fixations per trial throughout the exposure phase was investigated using an LMM. The difference estimate between the delay conditions was nonsignificant, β = –0.07, *t*(30) = –0.94, *p* = 0.356, suggesting no difference in the average number of fixations per trial between the two delay conditions. The slope estimate for the block number was also nonsignificant, β = –0.004, *t*(30) = –1.93, *p* = 0.063, showing no change as the block number increased. There was also no evidence of an interaction, β = 0.001, *t*(30) = 0.46, *p* = 0.652. The change in the average number of fixations per trial from pre-exposure to post-exposure was investigated using a 2 × 2 ANOVA. The main effect of the phase was nonsignificant, *F*(1, 30) = 0.46, *p* = 0.504, *η*_p_² = 0.02, and the main effect of the delay condition was nonsignificant, *F*(1, 30) = 0.01, *p* = 0.917, *η*_p_² = 0.0003. The interaction was also nonsignificant, *F*(1, 30) = 1.81, *p* = 0.188, *η*_p_² = 0.06. There was no change in the average number of fixations per trial from pre-exposure to post-exposure for either delay condition.

## References

[bib1] Avraham, G., Leib, R., Pressman, A., Simo, L. S., Karniel, A., Shmuelof, L., ... Nisky, I. (2017). State-based delay representation and its transfer from a game of pong to reaching and tracking. *eNeuro,* 4(6), 10.1523/ENEURO.0179-17.2017. PMC5788056 29379875

[bib2] Beadle, S. C., Muth, E. R., & Pagano, C. C. (2021). Using head-mounted displays to examine adaptation and calibration under varying perturbations. *Displays,* 66, 101985, 10.1016/j.displa.2020.101985.

[bib3] Beech, S., Stanton Fraser, D., Corston-Petrie, A., Gower, A. P., & Gilchrist, I. D. (2024). How changes in the mean latency, jitter amplitude, and jitter frequency impact target acquisition performance. *ACM Transactions on Applied Perception,* 22(2), Article 8, 10.1145/3701984.

[bib4] Beznosyk, A., Quax, P., Coninx, K., & Lamotte, W. (2011). Influence of network delay and jitter on cooperation in multiplayer games. In *Proceedings of the 10th International Conference on Virtual Reality Continuum and Its Applications in Industry*. New York, NY USA: Association for Computing Machinery. (pp. 351–354), 10.1145/2087756.2087812.

[bib5] Botzer, L., & Karniel, A. (2013). Feedback and feedforward adaptation to visuomotor delay during reaching and slicing movements. *European Journal of Neuroscience,* 38(1), 2108–2123, 10.1111/ejn.12211. 23701418

[bib6] Burge, J., Ernst, M. O., & Banks, M. S. (2008). The statistical determinants of adaptation rate in human reaching. *Journal of Vision,* 8(4), 20, 10.1167/8.4.20. PMC2684526 18484859

[bib7] Cámara, C., de la Malla, C., López-Moliner, J., & Brenner, E. (2018). Eye movements in interception with delayed visual feedback. *Experimental Brain Research,* 236, 1837–1847, 10.1007/s00221-018-527-8. 29675715 PMC6010481

[bib8] Chapman, H. L., Eramudugolla, R., Gavrilescu, M., Strudwick, M. W., Loftus, A., Cunnington, R., & Mattingley, J. B. (2010). Neural mechanisms underlying spatial realignment during adaptation to optical wedge prisms. *Neuropsychologia,* 48(9), 2595–2601, 10.1016/j.neuropsychologia.2010.05.006. 20457170

[bib9] Cieślik, K., & Łopatka, M. J. (2022). Research on speed and acceleration of hand movements as command signals for anthropomorphic manipulators as a master-slave system. *Applied Sciences,* 12(8), 3863, 10.3390/app12083863.

[bib10] Claypool, M., & Claypool, K. (2006). Latency and player actions in online games. *Communications of the ACM,* 49(11), 40–45, 10.1145/1167838.1167860.

[bib11] Cross, K. P., Cluff, T., Takei, T., & Scott, S. H. (2019). Visual feedback processing of the limb involves two distinct phases. *Journal of Neuroscience,* 39(34), 6751–6765, 10.1523/JNEUROSCI.3112-18.2019. 31308095 PMC6703887

[bib12] Cunningham, D. W., Billock, V. A., & Tsou, B. H. (2001). Sensorimotor adaptation to violations of temporal contiguity. *Psychological Science,* 12(6), 532–535, 10.1111/1467-9280.d01-17. 11760144

[bib13] Cunningham, D. W., Chatziastros, A., Von der Heyde, M., & Bülthoff, H. H. (2001). Driving in the future: Temporal visuomotor adaptation and generalization. *Journal of Vision,* 1(2), 3, 10.1167/1.2.3. 12678604

[bib14] Davis, J., Smyth, C., & McDowell, K. (2010). The effects of time lag on driving performance and a possible mitigation. *IEEE Transactions on Robotics,* 26(3), 590–593, 10.1109/TRO.2010.2046695.

[bib15] de la Malla, C., López-Moliner, J., & Brenner, E. (2012). Seeing the last part of a hitting movement is enough to adapt to a temporal delay. *Journal of Vision,* 12(10), 4, 10.1167/12.10.4. 22961221

[bib16] de la Malla, C., López-Moliner, J., & Brenner, E. (2014). Dealing with delays does not transfer across sensorimotor tasks. *Journal of Vision,* 14(12), 8, 10.1167/14.12.8. 25301016

[bib17] Ferrell, W. R. (1965). Remote manipulation with transmission delay. *IEEE Transactions on Human Factors in Electronics,* (1), 24–32, 10.1109/THFE.1965.6591253.

[bib18] Foulkes, A. J. M., & Miall, R. C. (2000). Adaptation to visual feedback delays in a human manual tracking task. *Experimental Brain Research,* 131, 101–110, 10.1007/s002219900286. 10759175

[bib19] Hadjiosif, A. M., Abraham, G., Ranjan, T., & Smith, M. A. (2024). Subtle visual latency can profoundly impair implicit sensorimotor learning. *bioRxiv,* 2024–03, 10.1101/2024.03.14.585093. PMC12062458 40341213

[bib20] Hosseini, E. A., Nguyen, K. P., & Joiner, W. M. (2017). The decay of motor adaptation to novel movement dynamics reveals an asymmetry in the stability of motion state-dependent learning. *PLoS Computational Biology,* 13(5), e1005492, 10.1371/journal.pcbi.1005492. 28481891 PMC5440062

[bib21] JASP Team. (2023). JASP (Version 0.17.2).

[bib22] Kennedy, J. S., Buehner, M. J., & Rushton, S. K. (2009). Adaptation to sensory-motor temporal misalignment: Instrumental or perceptual learning? *Quarterly Journal of Experimental Psychology,* 62(3), 453–469, 10.1080/17470210801985235. 18609410

[bib23] Kleiner, M., Brainard, D., Pelli, D., Ingling, A., Murray, R., & Broussard, C. (2007). What's new in Psychtoolbox-3. *Perception,* 36(14), 1–16

[bib24] Knelange, E. B., & López-Moliner, J. (2019). Decreased temporal sensorimotor adaptation due to perturbation-induced measurement noise. *Frontiers in Human Neuroscience,* 13, 46, 10.3389/fnhum.2019.00046. 30837854 PMC6382734

[bib25] Liu, R., Kwak, D., Devarakonda, S., Bekris, K., & Iftode, L. (2017). Investigating remote driving over the LTE network. In *Proceedings of the 9th International Conference on Automotive User Interfaces and Interactive Vehicular Applications*. New York, NY USA: Association for Computing Machinery. (pp. 264–269), 10.1145/3122986.312300.

[bib26] MATLAB. (2021). Version 9.11.0.1837725 (R2021b). Natick, MA: The MathWorks, https://www.mathworks.com.

[bib27] Mazzoni, P., & Krakauer, J. W. (2006). An implicit plan overrides an explicit strategy during visuomotor adaptation. *Journal of Neuroscience,* 26(14), 3642–3645, 10.1523/JNEUROSCI.5317-05.2006. 16597717 PMC6674132

[bib28] Messier, J., & Kalaska, J. F. (1999). Comparison of variability of initial kinematics and endpoints of reaching movements. *Experimental Brain Research,* 125, 139–152, 10.1007/s002210050669. 10204767

[bib29] Nagasaki, H. (1989). Asymmetric velocity and acceleration profiles of human arm movements. *Experimental Brain Research,* 74, 319–326, 10.1007/BF00248865. 2924852

[bib30] Newell, K. M., Liu, Y. T., & Mayer-Kress, G. (2001). Time scales in motor learning and development. *Psychological Review,* 108(1), 57, 10.1037/0033-295X.108.1.57. 11212633

[bib31] Rashid, U., Kumari, N., Signal, N., Taylor, D., & Vandal, A. C. (2020). On nonlinear regression for trends in split-belt treadmill training. *Brain Sciences,* 10(10), 737, 10.3390/brainsci10100737. 33066492 PMC7602156

[bib32] Rohde, M., & Ernst, M. O. (2013). To lead and to lag–forward and backward recalibration of perceived visuo-motor simultaneity. *Frontiers in Psychology,* 3, 599, 10.3389/fpsyg.2012.00599. 23346063 PMC3551234

[bib33] Rohde, M., & Ernst, M. O. (2016). Time, agency, and sensory feedback delays during action. *Current Opinion in Behavioral Sciences,* 8, 193–199, 10.1016/j.cobeha.2016.02.029.

[bib34] Rohde, M., Van Dam, L. C., & Ernst, M. O. (2014). Predictability is necessary for closed-loop visual feedback delay adaptation. *Journal of Vision,* 14(3), 4, 10.1167/14.3.4. 24599942

[bib35] Sheridan, T. B. (1993). Space teleoperation through time delay: Review and prognosis*.* *IEEE Transactions on Robotics and Automation,* 9(5), 592–606, 10.1109/70.258052.

[bib36] Stetson, C., Cui, X., Montague, P. R., & Eagleman, D. M. (2006). Motor-sensory recalibration leads to an illusory reversal of action and sensation. *Neuron,* 51(5), 651–659, 10.1016/j.neuron.2006.08.006. 16950162

[bib37] Waltemate, T., Senna, I., Hülsmann, F., Rohde, M., Kopp, S., Ernst, M., & Botsch, M. (2016). The impact of latency on perceptual judgments and motor performance in closed-loop interaction in virtual reality. In *Proceedings of the 22nd ACM Conference on Virtual Reality Software and Technology*. New York, NY USA: Association for Computing Machinery. (pp. 27–35), 10.1145/2993369.2993381.

[bib38] Wei, K., & Kording, K. (2009). Relevance of error: What drives motor adaptation? *Journal of Neurophysiology,* 101(2), 655–664, 10.1152/jn.90545.2008. 19019979 PMC2657056

[bib39] Wei, K., & Körding, K. (2010). Uncertainty of feedback and state estimation determines the speed of motor adaptation. *Frontiers in Computational Neuroscience,* 4, 1151, 10.3389/fncom.2010.00011. PMC2871692 20485466

[bib40] Wright, W. G. (2014). Using virtual reality to augment perception, enhance sensorimotor adaptation, and change our minds. *Frontiers in Systems Neuroscience,* 8, 56, 10.3389/fnsys.2014.00056. 24782724 PMC3986528

